# Brief peer coaching complements daily digital messages for chronic disease prevention among young adult Latinas

**DOI:** 10.1093/tbm/ibad036

**Published:** 2023-06-20

**Authors:** Kelly L’Engle, Evelin Trejo, Adam Landeros, Erika Zúñiga Sandoval, Jazmin Jauregui, Susan Yang

**Affiliations:** School of Nursing and Health Professions, University of San Francisco, San Francisco, CA, USA; Department of Hematology Oncology, Zuckerberg San Francisco General Hospital, University of California, San Francisco, San Francisco, CA, USA; School of Nursing and Health Professions, University of San Francisco, San Francisco, CA, USA; School of Nursing and Health Professions, University of San Francisco, San Francisco, CA, USA; School of Nursing and Health Professions, University of San Francisco, San Francisco, CA, USA; School of Nursing and Health Professions, University of San Francisco, San Francisco, CA, USA

**Keywords:** Latina, Chronic disease, Health coaching, Text messaging, Digital intervention

## Abstract

Young Latinas face multiple health challenges that place them at high risk for chronic diseases. Digital health promotion interventions can offer education and support to activate self-care and preventive behaviors. This pilot study evaluated a brief, theory-informed, culturally tailored intervention, *Examen Tu Salud*, that provided daily text and multimedia messages and weekly peer coaching via videoconference to improve health behaviors among young adult Latina women. Thirty-four participants who self-identified as Latina, female, and 18–29 years old were recruited from an urban college in Northern California to participate in a brief pilot test of the new intervention. Paired sample *T*-tests assessed health behavior and health activation changes from baseline to 1 month follow-up. Program participation and satisfaction were analyzed to assess feasibility of the intervention. Among 31 participants (91% completion), there were medium to large improvements in health outcomes. Confidence in preventing and managing one’s health (*t*[30] = 5.18, *p* < .001, *d* = 0.93), days of moderate-intensity physical activity (*t*[30] = 3.50, *p* < .001, *d* = 0.63), and fruit (*t*[30] = 3.32, *p* = .001, *d* = 0.60) and vegetable (*t*[30] = 2.04, *p* = .025, *d* = 0.37) consumption in a typical day increased. Intervention satisfaction and engagement with health coaches was high. We found that a brief digital coaching intervention designed for young adult Latinas has the potential to improve health activation and behaviors. More attention is needed to prevent chronic conditions among a growing number of Latinos in the USA.

**Implications**

**Practice:** Health promotion using remote peer coaching plus digital text and multimedia messages can provide tailored interventions for health behavior change to young adult Latinas.

**Policy:** Policy makers should implement culturally tailored primary prevention interventions using digital health promotion to reduce risk for chronic diseases among Latino communities.

**Research:** Future research is needed to test the effectiveness of multi-component digital health promotion approaches to improve health among at-risk young adults.

## INTRODUCTION

Chronic diseases are prevalent among Latinos in the USA. Approximately 4 in 10 people are estimated to be prediabetic, with both diabetes and prediabetes disproportionately affecting Latinos and other communities of color [1, 2]. The leading causes of death among Latinos include heart disease, cancer, stroke, and diabetes. Health behaviors that contribute to these illnesses are more prevalent among Latinos, who have higher rates of obesity and lower rates of physical activity than most other groups [3, 4]. Simultaneously, many Latinos face financial, cultural, geographic, and linguistic barriers to accessing high-quality primary and preventive care, with limited culturally sensitive interventions, a shortage of Latino health care providers, and lower health literacy contributing to health challenges [5, 6, 7].

The Latino population in the USA is relatively young, large, and growing. Nationwide, Latinos are the largest racial/ethnic minority group, and by 2060 more than half of Californians will be of Latino descent [8]. Latinos are substantially younger than other groups and represent the fastest growing minority group of youth in the USA [9]. Overweight and obesity along with diabetes and prediabetes have been increasing for many years among Latino youth [2, 6]. Acculturation—the process of adopting values and norms of the prevailing culture—helps to explain increased obesity rates among Latinos. Latinos who are born in the USA tend to have higher body weight in part due to acculturation [6] that results in adoption of a higher fat diet with more processed foods and sugar, in comparison to less acculturated or foreign-born Latinos who consume more fiber and less saturated fat [7, 10]. Latino young people have the highest rates of obesity among all subgroups in the USA [6, 7] and they have the highest rates of non-alcoholic fatty liver disease, an emerging risk factor for metabolic disease, compared to other youth [4]. Body mass index (BMI) is especially elevated for young Latinas who have higher risk for diabetes and obesity than males [4, 10]. Latina females also are less likely to engage in leisure-time physical activity and Latina adolescents are less likely to engage in daily physical activity compared to Latino males [3]. Furthermore, pregnant Latinas have high rates of gestational diabetes and pre-eclampsia [5, 10], which may contribute to a cycle of generational risk for diabetes and cardiovascular disease within families [5]. The growing population of young Latinas has numerous and increasing risk factors for chronic diseases [5].

Not nearly enough is being done to prevent chronic diseases among Latinas [4, 5, 11]. Culturally relevant and technology-supported preventive health interventions that can be delivered remotely offer Latino communities a promising strategy to improve health behaviors and reduce the burden of chronic diseases in later years [11, 12, 13, 14]. Research shows that mobile phone use among Latinos is as high or higher than other groups, and that mobile health education is a more common use of cell phones among Latinos [8, 15]. Digital health interventions have grown rapidly in popularity, yet few preventive interventions specifically target Latinos even though they may be especially successful for recruiting, engaging, and improving health behaviors among minorities [7, 11, 12]. Text messaging programs improve behavior across a variety of topic areas and population groups [16, 17]. However, intervention effect sizes range from small to moderate; for example, a meta-analysis of 35 experimental studies revealed that text messaging interventions had a small, positive, significant effect (*d* = 0.24, 95% CI 0.16, 0.32, *p* < .001) on preventive behaviors such as weight loss, physical activity, and smoking cessation [16]. Multi-component interventions tend to be more successful than text messaging-only interventions [11, 13, 16, 18] and increased personalization, tailoring, and interpersonal interaction may increase program impact [13, 18, 19].

Digital interventions that combine personalized health coaching with culturally relevant digital messages and support for behavior change have recently been implemented to tackle chronic diseases [20, 21, 22]. It has been shown to improve healthy behaviors and lifestyles across a range of populations and chronic disease-related health outcomes [23, 24, 25]. Remotely delivered coaching retains the key elements of behavioral counseling while increasing access, flexibility, interactions, and sharing of resources [22, 23, 26]. Especially for Latinos, digital interventions that offer a higher-touch human connection via coaching along with automated text messages may be most effective for behavior change [11, 13, 21, 27, 28].

In this one group pretest–posttest pilot study, we aimed to assess whether a culturally tailored, theory-based, brief intervention combining remote peer coaching with text and multimedia messages would improve health behaviors and be acceptable to young adult Latina women. The intervention, termed *Examen Tu Salud*, aimed to support 18–29 year olds in developing and achieving health goals, gaining positive behavioral skills, and improving their overall health. While targeting young adult male Latinos through intervention research also is critical, limited resources for targeted message development and matched-gender peer coaching guided our eligibility criteria for this pilot study.

## METHODS

### Participant recruitment

Participants were recruited via electronic communications between January and March 2021. We recruited graduate and undergraduate students who were enrolled at an urban university in Northern California (which did not meet in-person during this time due to the COVID-19 pandemic). Electronic flyers promoting the study were sent to Latino-focused student organizations to share with their members and included in newsletters from the college health service. A promotional email was sent to currently enrolled students who were identified as Latina in the university’s student database. A lottery for three $75 gift cards was offered as an incentive for participation. A study email was provided for interested students.

Eligibility criteria were as follows: self-identified as Latina, self-identified as female, between 18 and 29 years old, and owned a mobile phone (Apple or Android). Given the high rate of chronic conditions among Latinos and the high likelihood of developing chronic conditions in later years, we kept the recruitment criteria broad and inclusive.

### Study procedures

We used a one group pretest–posttest study design to assess potential impact on physical activity, healthy eating, and confidence managing one’s health over the 1-month study period. The Institutional Review Board at the university approved all study procedures.

A total of 55 individuals contacted the study email, and 14 of these were deemed ineligible because they were not Latina or outside of age limits. Thirty-four volunteers (83% of eligible individuals) were sent and signed the informed consent form and were enrolled into the study.

After providing informed consent, a link to the baseline electronic survey was emailed to each participant. Health coaches were assigned and contacted each participant via email to set up videoconference introduction meetings and to schedule four weekly coaching sessions. Simultaneously, each participant was enrolled into the text messaging platform (Simple Texting, Miami Beach, FL) and began receiving daily messages. A link to the follow-up electronic survey was sent to each participant 1 week after the final coaching session, which also included questions to gauge program satisfaction and feedback for improvement.

### Intervention

*Examen Tu Salud* is a culturally tailored peer health and wellness coaching program for young adult Latinas that was piloted in the Spring of 2021. Social Cognitive Theory [29] and the Transtheoretical Model [30] provided the overarching intervention framework for the peer coaching sessions along with daily digital health messages. Coaching was designed to complement and amplify motivational and skills-based text and multimedia messages. Weekly objectives for coaching also paired closely with the digital messages (Table 1). Health activation, a global construct that encompasses knowledge, skills, and self-efficacy for actively managing one’s health, was a main focus of the intervention [31]. Confidence for managing one’s health increases as one experiences small successes and moves along the behavior change continuum, with higher activation linked to a healthier diet, regular exercise, better management of chronic disease, and more persistent interactions with the healthcare system [31, 32]. Therefore, encouraging young adult Latina women to take an active role in managing their health may be an effective method of health promotion and disease prevention as well as supporting effective management of current or future chronic conditions.

**Table 1:** Weekly coaching objectives and example digital messages for the *Examen Tu Salud* intervention, mapped to behavior change strategies

Week	Theory and behavior change techniques	Coaching objectives and questions	Example digital messages
1—Goal Setting	Motivational interviewing Setting SMART goals	1. What do you want to work on? 2. Why is this important to you? 3. What do you hope to gain from the program? 4. How have the digital messages motivated you?	¿Sabías qué? Eating healthy, exercise, and being a healthy weight can add 7 years to your life AND it can cut your risk of type 2 diabetes in half. Hugs and health, *Examen* Hola Amiga! It’s important to know your “why.” Remind yourself every day why you want to eat healthy and stay active. Talk more about this with your coach.
2—Knowledge and Skills	Enhancing knowledge Developing skills Emphasizing benefits Highlighting successes Increasing self-efficacy	1. What did you do to meet your Week 1 goals? 2. What was something positive that happened regarding this goal? 3. Since the last session, what skills or behaviors have you been able to incorporate into your life? 4. How have the digital messages inspired you to take action this week?	*Examen* Advice: To be an active person, get 30 minutes of physical activity every day! Use your phone alarm as a reminder to get up and stay active. ¡Con ganas se puede! What did you do for your health today? Remember the goal you set with your health coach! Every step you take gets you to better health & happiness. ¡Con ánimo!
3—Overcoming Barriers	Monitoring behavior Reflecting Problem solving Increasing self-efficacy	1. What challenges have you faced in meeting your goals? 2. What can you do to address those challenges? 3. How can we modify your goal to make it more achievable for you? What’s another way to achieve your goals? 4. How have the digital messages helped you to overcome challenges this week? 5. What has gone well and why?	Adulting is hard but eating your fruits and veggies should not have to be. It is easy to add veggies to your day - put them in soups, salads or enjoy as sides! What’s something you did this week para mejorar tu salud [better your health]? Keep trying new foods, recipes, activities, and calming practices. Estamos contigo, *Examen*
4—Maintenance	Highlighting successes Reviewing skills Planning for future	1. What digital messages stood out to you this week? 2. What skills have you learned that will help you maintain your future health? 3. In 6 months, how would you like to see your health improved? What steps will you take to get there? 4. How will you incorporate SMART goal setting and problem solving skills to improve your health and lifestyle?	Spinach and strawberries are in season soon! Go to a farmers market this week with your friends or family. Try other seasonal fruits & veggies. ¡Vaya a probarlo! Reflect on your *Examen* journey: what skills have you learned that will help you maintain your physical, spiritual, and mental health for the future?

### Peer health coaching

One introduction meeting and four weekly health coaching sessions lasting 30 min on average were conducted via videoconference, with the same coach conducting all sessions. Coaches texted or emailed participants between weekly meetings to briefly check in and share additional resources. Latina students or graduates from a Master of Public Health program with a concentration in behavioral health and who had experience providing health education and counseling to community members in varied settings served as health coaches.

Coaches used motivational interviewing skills to elicit motivations for change by utilizing open-ended questions, prompting reflection on barriers and facilitators, providing encouragement and affirmation, and summarizing key conversational points [33, 34]. They also set and evaluated goals with participants and assessed stage of readiness for health behavior change, which are common practices in health coaching [24, 25]. Coaches were trained to use a guide that was adapted from other health coaching curricula [35, 36]. An overarching session framework was developed to inform the structure of weekly coaching, which included goal setting, assessing confidence in achieving goals, identifying stages of behavior change, providing tailored feedback and strategies to increase self-efficacy, and guiding the participant through the next stage of behavior change [29, 30]. Weekly objectives were established to guide the conversation with particular questions that could be used during each session related to participant’s specific goals as well as general skills for behavior change (Table 1). After each session, the health coach completed a Google Form to document the session length and participant’s progress in meeting behavior change goals.

### Digital health messages

Automated text and multimedia messages were sent daily for 1 month. To inform message design, we conducted formative research in four focus groups with 18–29 year old Latinas (4–9 participants per group) recruited from locations in the study community. We learned that diabetes was reported in almost every participant’s family, along with concerns about overweight, nutrition, cancer, and stress, and culturally tailored and age-appropriate chronic disease resources were seen as limited. Participants wanted health information that felt like it was coming from a friend, culturally relevant language such as “Spanglish,” and depictions of people and situations that are highly recognizable by the Latino community. Initial text messages also were discussed, and a mobile phone intervention was viewed as an optimal way to reach young Latinas.

**Fig 1:**
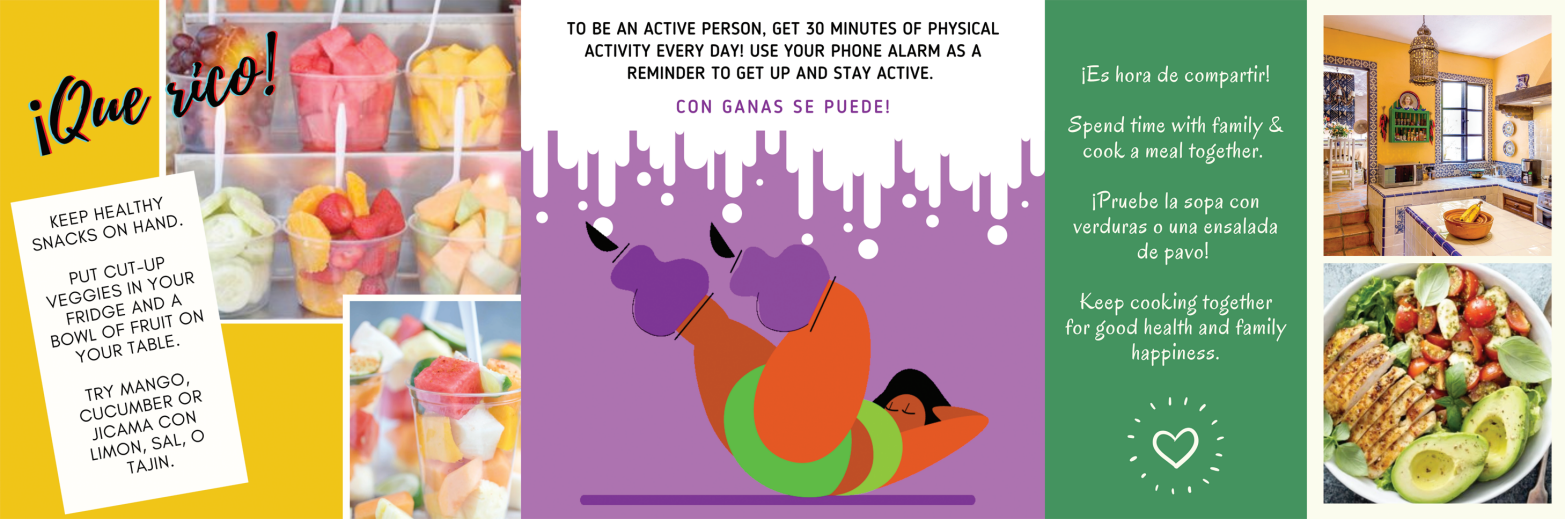
Examples of *Examen Tu Salud* multimedia messages culturally tailored to young adult Latinas

Messages were drafted by the ethnically diverse study team to focus on healthy eating, physical activity, and self-care (Table 1). Well known resources such as the National Diabetes Prevention Program [37] were consulted. Messages were designed to increase knowledge, skills, and self-efficacy for healthy behaviors and managing one’s health and to support processes of behavior change such as goal setting and monitoring, self-reevaluation, and stimulus control [29, 30]. The texts and images were culturally tailored: they featured familismo, Spanish terms, joyful emojis, body-positive images, and culturally relevant foods and practices (Fig. 1). Of 32 daily digital messages, 10 were multimedia messages with images and 13 included emojis for visual interest.

## MEASURES

### Background characteristics

Socio-demographic and other background variables were assessed at baseline and included age and gender, student status, marital status, current feelings about income, religious affiliation, and level of speaking Spanish. Participants were asked if they or anyone in their family had ever been diagnosed with diabetes and their height and weight which were used to compute BMI. Participants were asked to categorize themselves into one of five health lifestyle stages: not interested; thinking about it; planning for change within 30 days; made changes, but still having trouble following through; or had a healthy lifestyle for years.

### Health activation

Participants reported their general health (5-point scale from excellent to poor) and frequency of looking for health information (4-point scale from nearly every day to not at all) over the last seven days. Values were reverse-coded so higher scores indicate increasing health and information seeking.

Health activation was assessed with five items indicating a positive approach to managing one’s health and were adapted from the Patient Activation Measure [32]. Participants reported their agreement on a 5-point Likert scale that “I know how to prevent future health problems,” “I am confident I can figure out solutions when new situations or problems arise with my health,” “I am confident that I can take actions that will help prevent future problems with my health,” and “I am confident that I can maintain lifestyle changes like diet and exercise even during times of stress.” Participants also assessed their confidence about taking good care of one’s health (4-point scale from very confident to not confident at all). Items were summed and averaged to create the scale scores, which demonstrated acceptable reliability at baseline (α = 0.65) and follow-up (α = 0.75) with higher values indicating increased health activation.

### Health behaviors

Physical activity was assessed by asking participants how many days they did any moderate-intensity physical activity or exercise for at least 30 min and how many days they walked for at least 10 min [38, 39]. To assess nutrition, participants indicated how many cups of fruit and how many cups of vegetables they eat on a typical day [40]. We used brief assessments of physical activity and nutrition because our goal was to assess changes rather than make precise estimates about exercise duration and food intake. Self-efficacy for engaging in healthy behaviors (4-point scale from very confident to not confident at all) and intention to engage in healthy behaviors in the next month (4-point scale from likely to not at all likely) also were assessed. Values were reverse-coded so higher scores indicate increasing self-efficacy for healthy eating and regular physical activity.

### Program participation and satisfaction

We extracted participant attendance data and duration of each coaching session from the Google forms completed by health coaches after conducting each coaching session. To assess program satisfaction, participants were asked at the end of the follow-up electronic survey if they would recommend the *Examen Tu Salud* program to others (5-point scale from definitely yes to definitely no), and for open-ended feedback on what they liked about the program and recommendations for program improvement.

### Data analysis

Data analyses were conducted using SPSS v27 (IBM Corp, Armonk, NY). Descriptive statistics, including means and frequencies, were used to summarize participant characteristics at baseline for the enrolled sample (*n* = 34). Differences from baseline to follow-up for the 31 participants who completed the follow-up survey were evaluated using paired sample *T*-tests. Confidence intervals and effect sizes (Cohen’s *d*) for standardized mean changes in health behavior from baseline to follow-up were calculated. Following convention [41], effect sizes around 0.2 were considered small, 0.5 were moderate, and 0.8 were large. For all analyses, statistical significance was defined as *p* < .05; *p* < .10 findings are also reported for descriptive purposes given the early status of this research.

We conducted a thematic analysis of open-ended program satisfaction data by extracting the main aspects of the program that were most liked by participants and the main areas recommended for program improvement.

## RESULTS

### Sample characteristics

Sample characteristics at baseline are presented in Table 2. By design, participants were 18–29 years old (mean = 24.3, standard deviation = 3.1). Most were unmarried, identified themselves as Catholic, and spoke Spanish well. The large majority of participants (70%) reported they or a family member had diabetes. Of those reporting height and weight, two-thirds (66%) were classified as overweight or obese according to CDC guidelines, and the mean BMI was 27.7. At baseline, 10 respondents (29%) reported that they were thinking about or planning to make changes toward better health, while 24 respondents (71%) stated that they were actively working toward a healthier lifestyle.

**Table 2:** Baseline characteristics of Latina participants (*N* = 34)

	*N* (%)
Age categories	
18–24	16 (47.1)
25–29	18 (52.9)
Student status	
Undergraduate	13 (38.2)
Graduate	21 (61.7)
Never been married	30 (88.2)
Finds it difficult to live on present income	8 (23.5)
Catholic	23 (67.7)
Speaks Spanish well	27 (79.4)
Diabetes diagnosis for self or family member	23 (69.7)
BMI	
Healthy weight (*BMI <25 kg/m*^*2*^)	10 (29.4)
Overweight (*BMI ≥25 kg/m*^*2*^,<30 *kg/m*^*2*^)	13 (38.2)
Obese (*BMI ≥30 kg/m*^*2*^)	6 (17.6)
Did not report	5 (14.7)
Health stage	
Not interested in pursuing a healthy lifestyle	--
Thinking about making changes	7 (20.6)
Planning on making changes within 30 days	3 (8.8)
Some changes but trouble following through	22 (64.7)
Has had a healthy lifestyle for years	2 (5.9)

Enrolled study participants were split between undergraduate (47%) and graduate students (53%); three undergraduate students enrolled at baseline did not complete the follow-up survey.

### Changes in study outcomes

Paired sample *t*-tests showed statistically significant changes from baseline to follow-up on most outcome measures. Results showed large increases in health activation (*t*[30)] = 5.18, *p* < .001, *d* = 0.93). Moderate improvements in self-reported health (*t*[30] = 3.06, *p* = .002, *d* = 0.55) and increased information-seeking (*t*[30] = 2.19, *p* = .020, *d* = 0.39) were observed (Table 3).

**Table 3:** Changes in general health and health activation from baseline to follow-up

	*N*	Baseline Mean (*SD*)	Follow-up Mean (*SD*)	Mean change (95% CI)	*d*
Self-reported health	31	2.71 (0.69)	3.29 (0.69)	0.58 (0.19, 0.97)**	*0.55*
Health information-seeking	31	2.16 (0.78)	2.48 (0.68)	0.32 (0.02, 0.63)*	0.39
Health activation scale	31	3.29 (0.46)	3.77 (0.44)	0.48 (0.29, 0.67)***	*0.93*

*p< .05, **p < .01, ***p < .001.

Statistically significant improvements were observed from baseline to follow-up in physical activity and healthy eating skills and behaviors (Table 4). Days of moderately intensive physical activity (*t*[30] = 3.50, *p* < .001, *d* = 0.63) and walking (*t*[30] = 2.48, *p* = .010, *d* = 0.45) increased, along with self-efficacy (*t*[29] = 3.27, *p* = .001, *d* = 0.60) and intentions (*t*[30] = 3.76, *p* < .001, *d* = 0.68) to get regular exercise. Similar improvements were seen in moderately higher fruit intake (*t*[30] = 3.32, *p* = .001, *d* = 0.60) and vegetable consumption (*t*[30] = 2.04, *p* = .025, *d* = .37) during the study period. Moderate increases in intentions to eat more healthfully were observed (*t*[30] = 2.52, *p* = .009, *d* = 0.45), although self-efficacy for healthier eating increased only a small amount with marginal statistical significance (*t*[30] = 1.55, *p* = .066, *d* = 0.28).

**Table 4:** Changes in physical activity and nutrition from baseline to follow-up

	*N*	Baseline Mean (*SD*)	Follow-up Mean (*SD*)	Mean change (95% CI)	*d*
Days of moderate-intensity activity	31	3.84 (1.9)	4.87 (1.48)	1.03 (0.43, 1.64)***	*0.63*
Days of walking	31	5.55 (2.35)	6.26 (2.08)	0.71 (0.12, 1.30)*	*0.45*
Self-efficacy for exercising regularly	30	3.07 (0.83)	3.43 (0.73)	0.37 (0.14, 0.60)***	*0.60*
Intention to exercise regularly in next month	31	2.19 (0.70)	2.61 (0.50)	0.42 (0.19, 0.65)***	*0.68*
Cups of fruit in typical day	31	2.23 (0.72)	2.87 (0.92)	0.65 (0.24, 1.04)***	*0.60*
Cups of vegetables in typical day	31	2.35 (1.0)	2.74 (1.1)	0.39 (00, 0.77)*	*0.37*
Self-efficacy for eating healthfully	31	3.10 (0.83)	3.35 (0.66)	0.26 (−0.08, 0.60)^†^	*0.28*
Intention to eat healthfully in next month	31	2.26 (0.51)	2.55 (0.51)	0.29 (0.06, 0.53)**	*0.45*

^†^ *p* < .10, *p < .05, **p < .01, ***p < .001.

### Program participation and feedback

The large majority of scheduled coaching sessions (120/136 sessions, 88%) were completed. On average, coaching sessions lasted 31.5 min. All study participants said they would recommend the *Examen Tu Salud* program to others. Favorite aspects of the program included learning how to set goals that are achievable (“[My coach] was able to guide me in finding more attainable goals, that way I didn’t feel overwhelmed by anything and made it easier to start making little changes”) and accountability to the coach for meeting goals (“I really liked the weekly check-ins because they helped me with accountability. I was more motivated to complete my goals because I knew my coach would check-in on me.”). Many participants mentioned liking the culturally relevant digital messages and appreciating the tips and reminders (“The text messages and images were inclusive and targeted the Latinx women. The timing of the messages was great because it helped me stop and reflect at times, or other times it helped give me a reminder of my wellness goals”) and sharing a cultural background with the health coach (“Being able to talk to someone who shares a lot of the same cultural experiences with family and mental health. Also having that person be a support system for me.”).

Recommendations for program improvements included increasing the length from four to six to eight weeks, providing digital messages that were specific to the goals of each participant (“I would recommend a more personalized approach. Sometimes the messages I would receive weren’t relevant to what type of healthy [activity] I was looking for, and I would ignore the messages sometimes, but when one stood out to me I would take more time to look into it,”) and using more digital images (“I would recommend adding more visuals to the messages either through more emojis or photos because I noticed a difference in the intensity of my reaction to the message when it had a visual since I could easily remember it throughout my day.”)

## DISCUSSION

During the *Examen Tu Salud* pilot program, young adult Latina students reported meaningful and statistically significant improvements in their health from baseline to follow-up. Healthy eating, physical activity, and health activation improved, and data showed moderate to large effect sizes across study outcomes. Health coaching and daily text and multimedia messages were designed to consider young Latinas’ culture, needs, and values, and participants reported high engagement with the peer health coaches and culturally tailored messages. The combination of culturally relevant and technology-supported health promotion approaches, which are increasing in number [21, 28], may have been particularly impactful for empowering participants to activate and manage their health goals.

All three health coaches were Latinas, which may have been especially powerful at motivating and supporting behavior change. The coaches were able to understand the backgrounds and experiences of the Latina participants because they had similar experiences living with their own Latino families in the USA and navigating intersectional identities, social spaces, and health concerns. Community health workers who have similar cultural backgrounds and lived experiences as program participants are frequently employed to effectively deliver health programs to Latino communities [13, 27]. Trained peers capably deliver education and counseling from a sociocultural perspective that provides cultural tailoring of the intervention to the norms, values, and beliefs of participants [42] which improves health activation and intervention engagement and adherence [13, 27]. Furthermore, peers increase perceived social support and feelings of psychological safety, especially for younger people, as program participants learn new skills and problem solve, increase self-efficacy, and change their behaviors [43].

Digital health and wellness coaching is exciting for the increased access it provides [22, 23] along with the ability to tailor education and support to the cultural preferences [26] and health goals of participants [20, 26]. Simultaneously, text message interventions remain popular because they are accessible on all mobile phones regardless of brand, age, or features; they are scalable and low-cost; they support one and two-way interactions [28, 44] and they can support cultural tailoring [11]. Program engagement may have been higher because of the interactivity [13, 18, 26] and cultural tailoring [7, 27, 42]; intervention participants felt a personal connection to coaches and culturally tailored digital messages that may have led to increased health activation and larger effects on health behaviors [45]. Our *Examen Tu Salud* program aligns with recommendations that Latino-focused interventions always offer culturally tailored preventive education [7, 46] that maintains human interaction in technology-supported intervention delivery formats [13, 27].

Most digital health interventions enroll adults for secondary or tertiary disease prevention who already are connected to health care, and population-level primary prevention digital health interventions are limited. We intentionally enrolled all interested Latinas who were 18–29 years old because of their population-level increased risk for chronic diseases. Although it is likely that enrolled study participants chose to participate because of their knowledge of their own increased risk for chronic diseases, it is notable that 66% of study participants (19/29 who reported) were overweight or obese at baseline and an additional six with a healthy weight reported a diabetes diagnosis for themselves or a family member. Our findings of positive program impact are important because only a limited number of programs have proven effective for reducing overweight and preventing diabetes among younger Latinos [12, 14, 47] despite the high prevalence of obesity and increased risk of chronic diseases [1, 2, 4]. Since young Latinas are disproportionately at risk for poor health outcomes, more investigations of programs like *Examen Tu Salud* are essential to create a healthier future for Latino communities.

## LIMITATIONS

This study has several limitations. We used a single-group pre–post design, which weakens conclusions about program effectiveness due to the lack of a control group. Follow-up data collection occurred shortly after the final coaching session, so it is unknown whether changes were sustained over time, and self-reported health assessments may overestimate changes in study outcomes. This was a small pilot study of 34 Latino students enrolled in an institute of higher education at the undergraduate or graduate level, which limits generalizability of findings. However, we believe the digital health and wellness coaching approach can be appropriately designed and tailored for all ages, racial/ethnic groups, and socioeconomic levels [42, 46] due to interactive health coaching and tailoring of messages to match language preferences and cultural context of participants.

The brief *Examen Tu Salud* program was designed to be acceptable and feasible for busy young adults, but many participants wished the program was 2–4 weeks longer. While brief interventions require fewer resources and increase program access and feasibility, testing different lengths for program implementation is worthwhile to explore the optimum balance of feasibility and impact. Additionally, several participants stated that they would have preferred to receive digital messages specific to their individual health and wellness goals. In a review of technologies for chronic disease management among young people, codesign, and collaboration in developing the technological solution, along with tailoring technology to participants’ specific needs and context, were found to be essential for uptake and efficacy [48]. Although beyond the scope of this study, alternative technologies such as artificial intelligence (AI), chatbots, or other algorithm-based tools could be utilized for personalizing digital messages; this approach should be explored given that tailored and personalized messaging may increase effectiveness of digital interventions [11, 18, 26].

## CONCLUSION

Study results suggest that a brief, tailored digital health promotion intervention delivered remotely is an effective method for providing education and support for preventive health behaviors among young adult Latinas. Limited research has focused on young Latinas or explored how digital health and coaching programs interact with age and socioeconomic status to influence health and wellness among young Latinas. Greater focus on Latinas and digital health promotion interventions for more diverse Latino subgroups is recommended. Our findings support the feasibility and acceptability of adding personalized coaching to automated digital health interventions, and our data suggest that this combined approach may result in positive impacts on health behaviors and health activation for young adult Latinas. Future research should employ a counterfactual comparison group for a more robust evaluation and extend follow-up data collection to explore maintenance of behavior change and sustained impact on chronic disease risk factors over time.
